# Rationale and design of the RIACT–study: a multi-center placebo controlled double blind study to test the efficacy of RItuximab in Acute Cellular tubulointerstitial rejection with B-cell infiltrates in renal Transplant patients: study protocol for a randomized controlled trial

**DOI:** 10.1186/1745-6215-13-199

**Published:** 2012-10-26

**Authors:** Lena Schiffer, Mario Schiffer, Saskia Merkel, Anke Schwarz, Michael Mengel, Christopher Jürgens, Christoph Schroeder, Alexander A Zoerner, Kerstin Püllmann, Verena Bröcker, Jan U Becker, Maximilian E Dämmrich, Jana Träder, Anika Großhennig, Frank Biertz, Hermann Haller, Armin Koch, Wilfried Gwinner

**Affiliations:** 1 Department of Medicine/Nephrology, Hannover Medical School, Carl Neuberg Str. 1, Hannover, 30625, Germany; 2 Department of Biostatistics, Hannover Medical School, Carl Neuberg Str. 1, Hannover, 30625, Germany; 3 Alberta Transplant Applied Genomics Centre, University of Alberta, Alberta, Canada; 4 Department of Pharmacy, Hannover Medical School, Carl Neuberg Str. 1, Hannover, 30625, Germany; 5 Institute for Clinical Pharmacology, Hannover Medical School, Carl Neuberg Str. 1, Hannover, 30625, Germany; 6 Department of Hematology and Oncology, Hannover Medical School, Carl Neuberg Str. 1, Hannover, 30625, Germany; 7 Department of Pathology, Hannover Medical School, Carl Neuberg Str. 1, Hannover, 30625, Germany

**Keywords:** Cellular kidney allograft rejection, B-cells, Rituximab

## Abstract

**Background:**

Acute kidney allograft rejection is a major cause for declining graft function and has a negative impact on the long-term graft survival. The majority (90%) of acute rejections are T-cell mediated and, therefore, the anti-rejection therapy targets T-cell-mediated mechanisms of the rejection process. However, there is increasing evidence that intragraft B-cells are also important in the T-cell-mediated rejections. First, a significant proportion of patients with acute T-cell-mediated rejection have B-cells present in the infiltrates. Second, the outcome of these patients is inferior, which has been related to an inferior response to the conventional anti-rejection therapy. Third, treatment of these patients with an anti-CD20 antibody (rituximab) improves the allograft outcome as reported in single case observations and in one small study. Despite the promise of these observations, solid evidence is required before incorporating this treatment option into a general treatment recommendation.

**Methods/Design:**

The RIACT study is designed as a randomized, double-blind, placebo-controlled, parallel group multicenter Phase III study. The study examines whether rituximab, in addition to the standard treatment with steroid-boli, leads to an improved one-year kidney allograft function, compared to the standard treatment alone in patients with acute T-cell mediated tubulointerstitial rejection and significant B-cell infiltrates in their biopsies. A total of 180 patients will be recruited.

**Discussion:**

It is important to clarify the relevance of anti-B cell targeting in T-cell mediated rejection and answer the question whether this novel concept should be incorporated in the conventional anti-rejection therapy.

**Trial registration:**

Clinical trials gov. number: NCT01117662

## Background

High dose steroid therapy is standard-of-care for treating acute T-cell mediated rejection of renal allografts, along with intensified maintenance immunosuppressive therapy in some cases (KDIGO-Guidelines [1]). Only a minority of patients (20%) appears to be non-responsive to this treatment and may require additional treatments, for example, anti-thymocyte globulins [2]. The general perception is that successfully treated acute T-cell mediated rejections do not harm the graft significantly. Yet, in clinical experience not all patients will recover with full function after the anti-rejection treatment. Particularly late rejections, occurring later than six months after transplantation, may have detrimental effects on long-term renal function [3,4]. Even if renal allograft function returns to baseline values after anti-rejection therapy this may reflect some degree of compensatory hyperfiltration and obscure the loss of nephrons and development of interstitial fibrosis and tubular atrophy (IF/TA) [5]. In fact, several studies have established a relationship between progressive IF/TA and T-cell mediated acute rejection even in subclinical rejection cases (that is, without significant impairment of the glomerular filtration rate at the time of rejection diagnosis) [6,7].

Given these facts, the current treatment of acute T-cell mediated rejection may be suboptimal in many cases. One possible explanation could be that this type of rejection is more complex than reflected by current schemes and that therapy with steroids alone does not fully address this complexity. Recent studies demonstrated that more than 30% of the T-cell mediated tubulointerstitial rejections additionally contain a significant number of B-cells in the infiltrates [8]. The actual pathogenetic role of these B-cell infiltrates remains to be established but the first studies and our own data indicate an inferior one-year allograft function and graft survival in patients with T-cell mediated rejections with B-cell infiltrates [9-11]. Steroid therapy alone, which is primarily targeting T-cells, may not be adequate in these cases, raising the question of additional B-cell directed treatment strategies. Rituximab is a B-cell depleting antibody which is directed against the CD20 epitope and leads to apoptosis of mature B cells. It has been approved for treatment of diseases with B-cell activity, such as B cell lymphoma and rheumatoid arthritis [12]. Thus far, only one pilot study examined the effect of rituximab in the treatment of biopsy-proven acute T-cell mediated rejection with B-cell infiltrates [13]. In this randomized prospective study, 10 children with acute T-cell mediated rejection with B-cell infiltrates were treated with rituximab in addition to standard-of-care anti-rejection treatment (steroid boli and/or thymoglobuline). Compared with the control group of 10 children who received standard-of-care treatment only, rituximab-treated children had better recovery of allograft function (P = 0.026) and improved biopsy rejection scores (P <0.0001) at the follow-up biopsy after six months. Furthermore, a few case reports in adults suggest a benefit of rituximab alone or in combination with the usual anti-rejection treatment in patients with acute (therapy refractory) rejection [14,15].

Beneficial effects of targeting B cells in T-cell-mediated acute rejection with rituximab can be based on the concept that allospecific T-cell/B-cell cross-talk in secondary lymphatic organs. After migration into the allograft and into nodular/tertiary lymphatic organs, B-cells induce an increased antigen presentation and aggravated chemokine milieu [16]. This process may be halted by rituximab, as a single dose of rituximab at 375 mg/m^2^ has been shown to induce a sustained reduction of B-cells in the peripheral blood but also in the kidney allograft in most patients within the first 35 days [17].

Despite the promise of the cited studies [13,18] critical evaluation invariably leads to the conclusion that the evidence is not sufficient to treat acute transplant rejection with rituximab. This includes the small number of reported cases, short follow-up periods and heterogeneity of inclusion criteria, and the histological grading in these studies. Therefore, a double-blind, randomized, placebo-controlled trial with a sufficient number of patients and stringent histological defined inclusion criteria is needed.

These aspects are considered in the design of the RIACT-study. The acronym RIACT–study stands for a multi-center randomized placebo controlled double blind study to test the efficacy of RItuximab in Acute Cellular tubulointerstitial rejection with B-cell infiltrates in renal Transplant patients (RIACT).

## Design

### Study design

The RIACT-study is a randomized, double-blind, placebo-controlled, parallel group multicenter Phase III study. The study hypothesis is that treatment with rituximab in addition to the standard therapy with steroid-boli is superior to the standard therapy in acute T-cell mediated tubulointerstitial rejections in which B-cell infiltrates are present. The primary efficacy endpoint of the study is the change of the glomerular filtration rate (GFR) one year after intervention compared to the baseline GFR before the rejection episode.

Secondary endpoints are: 1) the progression of interstitial fibrosis and tubular atrophy (IF/TA) between the allograft biopsy that led to enrollment in the study and a scheduled protocol biopsy one year after intervention and 2) safety and side effect aspects of rituximab in kidney transplanted patients.

The study is recruiting a total of 180 patients with an acute T-cell mediated tubulointerstitial rejection who also show significant B-cell infiltrates in their biopsies, defined as >30 CD20+cells/hpf. After diagnosis of a B-cell-rich acute T-cell mediated tubulointerstitial rejection and informed consent (run-in phase), the patients will be centrally randomized for double-blinded treatment with rituximab or placebo, which will be given in addition to the standard anti-rejection therapy with steroid boli (study phase).

During the study phase a sufficient B-cell depletion (<5 B-cells/μl peripheral blood) after the first application of rituximab will be confirmed by fluorescence activated cell sorting (FACS) analysis. Patients with rituximab treatment and insufficient B-cell depletion undergo a second intervention with the same dose of rituximab. If a follow-up B-cell count in these patients reveals B-cells numbers above the target value, these patients will be excluded from the study. The follow-up period after intervention is 12 months. At this time, the primary endpoint renal allograft function change is determined, along with the secondary endpoints as safety parameters and degree of renal allograft fibrosis and tubular atrophy in a protocol biopsy.

**Figure 1:**
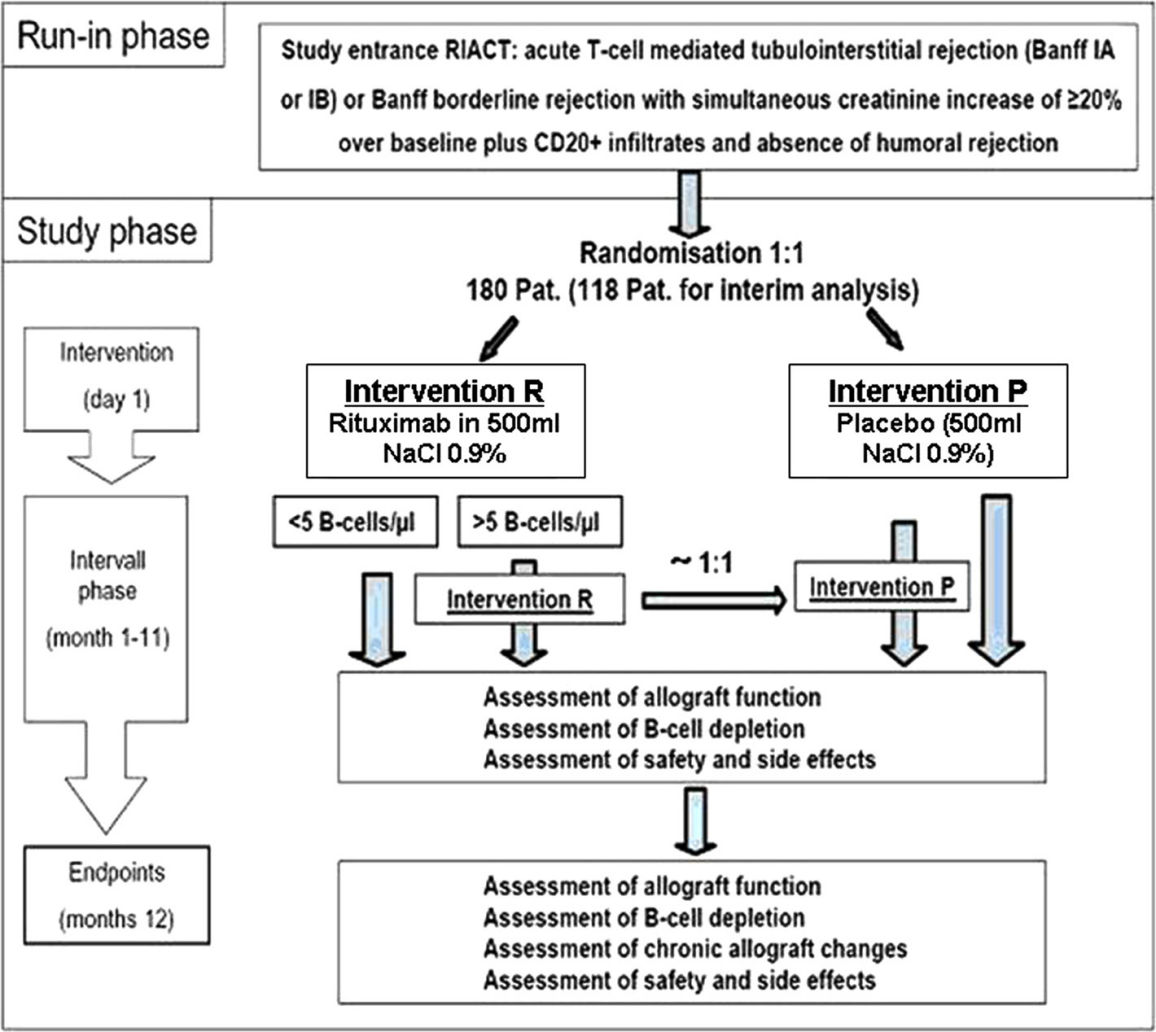
**Flowchart of the study design of the RIACT study.** Independent from the study protocol, all patients will receive steroid boli for rejection therapy.

The trial flow is outlined in Figure 1.

B-cell infiltrates in the biopsies are considered significant in the RIACT study, when more than X400 are found. However, the definition of significant B-cell infiltrates is very heterogeneous in previously published studies. The rationale for choosing 30 CD20+ cells /per hpf (X400) in our trial is based on the observations of our pilot study, which was also used for assessment of graft outcome and sample size calculation. Furthermore, the defined cell number lies within the limits reported in other published studies [19].

Patients with Banff IA, IB or with a Banff borderline rejection with a simultaneous increase in serum creatinine of ≥20% over baseline values before the borderline finding are eligible. The study will be carried out in Germany by, so far, 13 leading kidney transplant centers. The study design is illustrated in Figure 1.

For the study design we followed the guidelines “European legislation on orphan medicinal products (Regulation (EC) No 141/2000)” and the “Guideline on Clinical Trials in Small Populations (CHMP/EWP/83561/2005)”. The study protocol has been approved by the local ethics committees and national authorities. The trial is being conducted in accordance with national laws, good clinical practice (GCP)-guidelines and the Declaration of Helsinki.

### Inclusion and exclusion criteria

The population of renal allograft recipients is heterogeneous (for example, age, gender, first or repeated transplant, immunological risk), yet, all patients are at risk for acute rejection. Therefore, the inclusion criteria in the RIACT–trial are laid down in a way that allows as many patients as possible to participate in this study unless medical reasons advise against inclusion. Included are all female and male patients aged 18 years or older with the diagnosis of a T-cell mediated acute tubulointerstitial rejection (Banff IA/IB, or borderline rejection with simultaneous increase of creatinine of ≥20% over baseline creatinine before the borderline finding), and B-cell infiltrates, defined as more than 30 CD20+cells/hpf. All immunosuppressive regimens according to local transplant center standards at the time of rejection diagnosis are acceptable, except previous use of rituximab to have the obtained results applicable to a broad patient population. Patients with a humoral rejection (morphological signs of glomerulitis/capillaritis, positive C4d staining) and patients with positive SV40 staining as a sign of polyomavirus-infection are not eligible. The acute rejection must be diagnosed between 6 weeks and 12 months after kidney transplantation. The baseline allograft creatinine clearance (calculated by the abbreviated MDRD formula [20]; before the rejection has to be over 25 ml/min. At screening, women of child-bearing age must have a negative pregnancy test, must not be nursing and have to agree in using adequate contraception throughout the study.

Patients are not eligible for the study entry if they had previous adverse reactions against anti-CD20 antibodies, have received rituximab within 12 months prior to the planned inclusion in the RIACT study, have any active infections (for example, active CMV, HIV or replicative hepatitis B or C infection), or had a splenectomy. Reasons for exclusion also include malignant tumors, cardiac diseases, such as heart insufficiency (New York Heart Association (NYHA) III-IV) or severe arrhythmia (Table 1).

**Table 1:** Main inclusion and Exclusion criteria

	
Inclusion criteria	1. Kidney transplantation >6 weeks and <12 months before randomization
	2. Age ≥18 years
	3. Presence of an acute T-cell mediated tubulointerstitial rejection (Banff IA or IB) or Banff borderline rejection with simultaneous serum creatinine increase of ≥20% over baseline
	4. Significant numbers of B-cell infiltrates (defined as >30 CD20+cells /hpf)
	5. Absence of C4d and SV40
	6. Creatinine clearance >25 ml/min.
	7. Reliable contraception
	8. Written informed consent
Exclusion criteria	1. Known adverse reactions against rituximab or concomitant medication
	2. Application of rituximab for any reason 12 months prior inclusion
	3. Women who are pregnant or breastfeeding
	4. Active CMV, HIV, replicative Hepatitis B or C, or other relevant infections
	5. Splenectomy
	6. Heart insufficiency (NYHA III-IV)
	7. Severe arrhythmia
	8. Insufficiently controlled diabetes mellitus (HbA1c >10 %)
	9. Contraindication for a second transplant biopsy (for example, coagulation disorders)
	10. Other reasons according to the assessments of the study physician

### Sample size calculation

Sample size calculation is based on the t-test for independent samples. It is assumed that ANCOVA with adjustment will lead to an increase of the power in the final analyses. The type-I-error is set to 5% (two-sided) and the power is set to 80%. The sample-size calculation is based on the observations of 51 patients with acute T-cell mediated tubulointerstitial rejection that were treated at the Medical School Hannover between 2007 and 2009. A total of 27 patients without B-cell infiltration had a mean increase over the baseline function before the rejection in the GFR of 2.5 ml/min (± 24.5 standard deviation) one year after treatment of the rejection episode. In the 24 patients with B-cell infiltrates, a deterioration of GFR by minus 12 ml/min (± 27.8 standard deviation) was observed.

A conservative assumption is that the change in GFR over one year could be zero in the treatment group and could be −12 ml/min in the control group, which would require a sample size of 86 patients per group. In a more optimistic scenario it is assumed that an improvement of 2.5 ml/min can be achieved in the treatment group. In the control group, the same deterioration as in the conservative assumption (−12 ml/min) could be observed. In this scenario, 58 patients per treatment group would be necessary. Therefore, an interim analysis is planned as soon as the primary endpoint is reached in 116 patients. An interim analysis plan according to O’Brian and Flemming will be used [21]. The null hypothesis is that the change of the GFR is the same in patients treated with rituximab and under placebo. The null hypothesis is rejected in the interim analysis if the one-sided P-value is less than 0.005. In case the study is continued to the final analysis, the null hypothesis can be rejected if the P-value in the final analysis is less than 0.023 (one-sided). The application of this interim analysis increases the number of patients per treatment arm to 88 patients.

If the optimistic assumption for the sample size calculation holds true, the study has a chance of approximately 60% to reject the null hypothesis at the time of the interim analysis and thus ended at this point. In addition, the study could be terminated, with consultancy of the data monitoring committee, due to expected inefficacy.

Although all efforts will be done to achieve complete data for the primary endpoint for all patients, two additional patients will be recruited per treatment group to ascertain sufficient numbers of patients in each group. Thus, a total of 180 patients will be included into the trial. A recruitment period of three years is expected with 13 centers recruiting for the trial.

### Interventions and study plan

The diagnosis of an acute T-cell mediated tubulointerstitial rejection (Banff IA/IB) or a Banff borderline rejection with significant numbers of B-cells in the infiltrates is one of the major inclusion criteria for the RIACT study. To ensure a timely beginning of the anti-rejection therapy, the histopathological diagnosis will be made by the pathologists who are usually consulted by each participating center for kidney biopsy evaluation (all pathologists are in university departments). To achieve the highest degree of comparability and to reduce inter-center variability in the histopathological diagnosis, all pathologists will be provided with the same automated immunohistochemical staining platform (BondMaxTM, A. Menarini Diagnostics, Berlin, Germany). The stainings will be performed using the same stainer program and the same antibody- and staining-solutions. For the screening, the biopsies will be stained for CD20, C4d and SV40 in the above described fashion, in addition to the normal chemical stainings performed in each center for the rejection diagnosis.

All patients that fulfill the medical inclusion criteria and the histological inclusion criteria are asked to participate in the RIACT-study. After obtaining their written informed consent, patients attend a screening visit. At this visit the patients are asked to provide a medical history (with emphasis on the transplant history) and undergo a physical examination. Vital signs are measured and blood samples are taken to determine creatinine, cystatin C, electrolytes, liver enzymes and blood counts, including B-cell count by FACS-analysis. Furthermore, a urine status will be assessed (leukocytes, nitrite, protein, haemoglobin). To exclude pregnancy at the time of inclusion, ß-HCG-levels in female patients are measured. According to the standard therapy protocol for acute T-cell mediated tubulointerstitial rejection, all patients will receive the first, out of three, steroid bolus upon detection of B-cell infiltrates in the biopsy (defined as more than 30 CD20^+^-cells/hpf), patients enter the double-blind treatment period by randomization to treatment with either (A) standard treatment (two more steroid boli for the next two days) plus placebo (500 ml NaCl0,9%), or (B) standard treatment (steroid boli for the next two days) and the study drug, a single dose intravenous infusion of rituximab (375 mg/m^2^).

Both treatment groups will receive additional medication that is intended to prevent potential side effects of rituximab, consisting of antihistaminics and an antipyretic medication (paracetamol) together with the study medication. All patients will also receive prophylaxis for pneumocystis jirovecii pneumonia for six months after the anti-rejection treatment according to the local center schemes.

According to the literature, a single dose of rituximab at 375 mg/m^2^ is able to induce a sustained reduction of B-cells in the peripheral blood but also in the renal allograft in most patients within the first six weeks [17]. Following these findings and with the aim to keep the dosage of rituximab and, therefore, possible side effects as low as possible, rituximab will be given generally only once in the RIACT study. To assure a complete B-cell depletion in the peripheral blood (defined as <5 B-cells/μl), B-cell numbers in the peripheral blood will be assessed by FACS analysis on Day 35 ± 7.

Patients in the rituximab group with more than five B-cells/μl on Day 35 ± 7 will receive a second dose of rituximab (375 mg /m^2^). Accordingly, sufficient numbers of patients in the placebo group will be randomized in advance to receive a second round of placebo treatment. (For each patient in the placebo group it will be randomized in advance whether he will receive a second round of placebo treatment or not. If the rate of patients that need a second dose of rituximab is lower or higher than expected, the rate of patients that receive a second dose of placebo will be adapted.) In patients with a second treatment course, B-cells counts will be repeated on Day 72 ± 7. Patients in the verum group that continue to have more than five B-cells/μl in the peripheral blood will not receive further treatments with rituximab. Nevertheless, these non-responders will be followed at the regular study visits as well.

At the half-year visit after randomization (Day 182 ± 28), B-cell counts and laboratory tests will be repeated in all patients. One year after enrollment (Day 365 ± 28), final hematological and laboratory tests and a re-biopsy of the transplant will be performed (see Table 2). The progression of interstitial fibrosis and tubular atrophy ΔIF/TA between the biopsy that led to enrollment in the study and the biopsy one year after intervention will be quantified using specific staining und computerized image analysis.

**Table 2:** Study visits

	
Visit 1 (Day −1/-3 (prestudy))	-written consent
	-physical examination
	-laboratory tests
	-B-cell count
	-assessment of allograft function
	-beginning of rejection therapy (steroids) according to center standards
	-randomization
Visit 2 (Day 0)	-rituximab or placebo + standard concomitant medication
Visit 3 (Day 1)	- physical examination
	- patient history for specific side effects
Visit 4 (Day 35 ± 7)	- physical examination
	- laboratory tests
	- B-cell count
Visit 4a: if no complete B-cell depletion (<5/μl) is achieved:	second application of study medication or placebo (Visit 4a))
Visit 4b: (if study medication was administered in Visit 4a):	- physical examination
	- patient history for specific side effects
Visit 4c: (if study medication was administered in Visit 4a):	- physical examination
	- laboratory tests
	- B-cell count
Visit 5 (Day 182 ± 28)	- physical examination
	- laboratory tests
	- B-cell count
	- assessment of allograft function
Visit 6 (Day 356 ± 28)	- physical examination
	- laboratory tests
	- B-cell count
	- assessment of allograft function
	- re-biopsy

### Methods against bias

The RIACT study is a double-blind, randomized, placebo-controlled phase III trial. Randomization will be performed centrally stratified by study center. The allocation to treatment arms results from a permuted-block randomization with variable block length. FACS results, that could give a hint which patient is in the verum respectively in the placebo group, will not be handed out to the study physician, but will be sent from the FACS facility to the unblinded randomization team in the Department of Biostatistics. The study physician will get the information concerning which patient has to receive a second dose of medication from the randomization team.

The biopsy readings, including quantification of fibrosis, will be done without knowledge of the assignment to the treatment groups. All biopsies will be submitted to a second reading by a panel of renal pathologists. Biopsies with divergent diagnoses will be discussed by the pathology consortium to reach a final diagnosis.

The primary analyses will be conducted on the intention-to-treat (ITT) population. Patients without complete B-cell depletion will also be observed and assessed according to the study protocol.

The likelihood of drop-outs from the study is negligible as all patients are under continuous surveillance in the participating centers. Although some patients may wish to withdraw prematurely from the trial, it is assumed that GFR-values one year after inclusion into the study - the primary endpoint of the study - can be obtained from all patients. However, if the GFR one year after inclusion into the study cannot be assessed, the last known plausible GFR value will be used, because it is assumed that a critical clinical progress will be carefully monitored.

### Study assessments

The GFR of the renal allograft is the ultimate parameter that describes acute and chronic aspects of the graft function and is the most widely used measure for the outcome in renal transplantation studies. Sensitivity of the GFR can be improved by using the patients’ individual percentage change in GFR in a defined time interval (“delta GFR”). The primary objective is to show that additional anti-rejection treatment with rituximab is more efficacious than the standard anti-rejection treatment with regard to the allograft function one year after these treatments. For this purpose, individual changes in the glomerular filtration rate (GFR) will be determined using the baseline GFR before the rejection and the value at one year after rejection treatments. The GFR will be calculated using the simplified MDRD formula (eGFR = 186*(0.742 for females)*creatinine-1.153*age −0.203) [20].

As a second endpoint, interstitial fibrosis and tubular atrophy (IF/TA) will be used as an indicator of established chronic damage. To achieve maximum possible accuracy, biopsy specimens will be stained for fibrosis (Sirius Red) and quantified by computerized image analysis in specified areas of the renal tissue (renal cortex).

Again, to enhance the informational value of this parameter, the patient’s individual change of IF/TA in the initial biopsy at the time of acute rejection and inclusion in the study to the biopsy at the end of the study will be used (“delta-IF/TA”).

To assess the safety of the intervention, occurrence and frequency of adverse events in both treatment groups will be analyzed. These include the patient’s self-reported events and those inquired about by the study physician during regular study visits or obtained from additional in- and outpatient attendances. As a combined secondary safety endpoint, further rejection episodes, terminal graft failure during the observation period, severe infections and patient deaths will be analyzed.

### Ethics statement

The study was approved by the ethics committee of Hannover Medical School as the leading center and the Paul Ehrlich Institute, as well as by all local ethics committees of the participating centers.

### Statistical analysis

The primary analyses will be performed in all patients who have received a least one dose of the study medication. Sensitivity analyses will be performed in the per protocol population, including all patients that finish the study at the last visit one year after study entry. The change in GFR between baseline and the assessment one year after the rejection episode will be performed with ANCOVA analysis adjusted for baseline, treatment and center. One interim analysis and one final analysis will be performed in this trial following the aforementioned decision rules (see Sample size calculation).

As soon as it can be demonstrated that rituximab is superior to placebo with respect to the primary endpoint, the secondary endpoint progression of interstitial fibrosis and tubular atrophy will be assessed in a confirmatory sense. Increase of fibrotic and atrophic interstitial area is analyzed with ANCOVA with treatment, center and baseline IF/TA in the first biopsy included as co-variates. Subanalyses will include the effect of rituximab in patients with different severity grades of rejection (Borderline, Type I and II), different immunosupression before inclusion in the study and patient groups receiving one versus two applications of rituximab. Frequency of adverse events will be reported per treatment group and analyzed with a chi-square test. *P*-values will be assessed descriptively.

## Discussion

Acute T-cell mediated tubulointerstitial rejections occur in 15 to 30% of all transplanted patients. In approximately 30% of these cases, B-cell infiltrates are detectable, which may be associated with less beneficial transplant outcomes. However, the current standard therapy for acute rejections focuses on T-cells.

The RIACT study, therefore, tests the hypothesis that additional treatment of the rejection with rituximab is more efficacious than the standard treatment with steroid boli alone. This novel therapy will be studied in a multi-center randomized, placebo controlled, double blind trial with a sufficient number of patients. The recruitment of 180 eligible patients will be challenging despite the multi-centric approach with 13 participating centers. Should the recruitment rate prove to be lower than expected in the first six months after the study begins, more centers will be asked to participate. If this study proves that rituximab is beneficial in acute rejections that contain B-cell infiltrates with regard to the long-term allograft function and without a significant increase in adverse events, future protocols for the treatment of acute rejection most likely will have to incorporate the concept of B-cell targeting.

## Trial status

The study is recruiting patients.

## Abbreviations

FACS: Fluorescence activated cell sorting; GCP: Good clinical practice; GFR: Glomerular filtration rate; IF/TA: Interstitial fibrosis and tubular atrophy; ITT population: Intention-to-treat population; RIACT-study: RItuximab in Acute Cellular tubulointerstitial rejection with B-cell infiltrates in renal Transplant patients.

## Competing interest

The authors declare no conflicts of interest.

## Authors’ contributions

LS, WG, MS, SM, AS, MM and HH conceived the study. LS, WG, MS, HH, AG and AK designed the trial and obtained research funding. CS and AAZ are in charge of pharmacovigilance and prepared the data monitoring committee charter. KP manages the FACS analysis of the study. MD, VB, JB and JT will evaluate the allograft biopsies; MD developed a histological scoring system for the B-cell infiltrates. AG, BF and AK will undertake data analyses. LS, WG and MS wrote the first draft of this paper, which has been critically revised by all co-authors. All authors have read and approved the final version of the manuscript.

## Collaborators

F. Eitner, G. Kroll , A. Mühlfeld (Department of Nephrology, RWTH University Hospital, Aachen, Germany)

L. Rump, S. Kücükköylü, L. Sellin (Department of Nephrology, Medical Faculty, Heinrich-Heine University Düsseldorf, Germany)

M. Wiesener, K. Hilgers, K.-U. Eckardt (Department of Nephrology and Hypertension, Friedrich-Alexander-University,)

O. Witzke, (Department of Nephrology, University Hospital Essen, University Duisburg-Essen, Essen, Germany)

S. Zschiedrich, M. Geyer (Renal Division, University Hospital Freiburg, Freiburg, Germany)

V. Kliem, P. Weithofer, (Department of Internal Medicine and Nephrology, Nephrologisches Zentrum Niedersachsen, Hann. Muenden, Germany)

M. Schad, B. Suwelack, (University Hospital Muenster, Muenster, Germany).

C. Rüster, G. Wolf, (Department of Nephrology University-Hospital Jena, Jena, Germany)

T. Feldkamp, U. Kunzendorf, (Department of Nephrology and Hypertension, University Hospital Schleswig-Holstein, Campus Kiel, Kiel, Germany.)

T. Kisner, T. Benzing (Renal Division, Department of Medicine, University Hospital of Cologne, Cologne, Germany)

W. Arns , A. Huppertz (Clinic Cologne-Merheim, Cologne, Germany)

A. Habicht, M. Fischereder (Nephrologisches Zentrum, Medizinische Poliklinik Innenstadt, Klinikum der Ludwig-Maximilians-Universität München, München, Germany)
